# Health Tract, No. 215

**Published:** 1864-09

**Authors:** 


### VALUE OF FOOD.

Without strength or warmth we die; food imparts these, and is proportionably valuable; hence it is "nutritious," that is, nourishes, sustains, supports life The elements of food which do this are called carbon, yielding warmth, and nitrogen, yielding strength or flesh. Butter, fat, and oil are almost wholly carbon—contain no nitrogen—can not make flesh or give strength; on the other hand apricots, cherries, and peaches contain no carbon. A man who fed on them exclusively would freeze to death, would die for want of the warming part of nutriment. Meats give both warmth and strength, and so do most articles of food, but in varying proportions.

For those who work, that food is cheapest which, dollar's worth for dollar's worth, affords the most strength, the most power to labor. The investigations and experiments of Baron Liebig and others seem to show that one bushel of oats at sixty-eight cents a bushel, yields five pounds of the muscle, flesh, or strength element, costing thirteen cents per pound, while the same amount of "muscle," in the now common acceptation of the term, derived from roast beef, at twenty-five cents per pound at the butcher's stall, would cost two dollars and six cents! The Irish masses do not eat meat once a week, yet they work hard, live healthily, and when temperate, live long. The Scotch glory in oatmeal, and are a hardy race. One third of the human family live chiefly on rice.

It would be as healthful as economical for the industrious poor of our land to live chiefly on cereals, as wheat, corn, oats, rye, and barley, and when they can afford it, have fruits and berries, raw, ripe, and perfect in their natural state, as desserts.

| Articles. | Cost. | Muscle Element. yielded. | "Muscle" cost per pound. |
|---|---|---|---|
| Oats, | $0 68 per bushel, | 5 pounds, | 13 cents. |
| Peas, dried, | 2 00 " | 14 " | 14 " |
| Beans, " | 2 50 " | 16 " | 15 " |
| Corn, | 1 10 " | 6 " | 17 " |
| Barley, | 1 50 " | 8 " | 18 " |
| Turnips, | 50 " | 1 " | 41 " |
| Flour, unbolted, | 11 00 per barrel, | 25 " | 44 " |
| Flour, fine, | 12 00 " | 22 " | 54 " |
| Potatoes, | 1 50 per bushel, | 2 " | 94 " |
| Meats, 8½ pounds, | 25 per pound, | 1 " | 2 06 " |

